# Alterations of cognitive function and hippocampal metabolism in asymptomatic neurosyphilis patients: based on ^1^H-magnetic resonance spectroscopy

**DOI:** 10.3389/fneur.2026.1782928

**Published:** 2026-04-29

**Authors:** Jing Han, Yanrong Yuan, Jun Wang, Xiaokang Ni, Yongxing Yan

**Affiliations:** 1 Department of Neurology, Hangzhou Third People’s Hospital, Zhejiang, China; 2 Department of Psychiatry, Hangzhou Third People’s Hospital, Zhejiang, China

**Keywords:** asymptomatic neurosyphilis, cognition, hippocampus, magnetic resonance, spectroscopy, syphilis

## Abstract

**Objectives:**

Neurosyphilis has been found to be associated with cognitive decline. However, as a common type of neurosyphilis, the change of cognitive function in asymptomatic neurosyphilis (ANS) has received little attention. This study aims to investigate the cognitive function and cellular metabolism changes in bilateral hippocampus in ANS.

**Methods:**

Twenty-four ANS, 20 syphilis patients and 23 control individuals who were admitted in Hangzhou Third people’s Hospital from June 2021 to July 2025 were selected. ^1^H-MRS (magnetic resonance spectroscopy) was used to detect the cellular metabolism of bilateral hippocampus in these subjects (ANS patients detect before treatment and 6 months posttreatment). The Montreal cognitive assessment (MoCA) was also used to evaluate cognitive function of these patients. Compare the differences between three groups, and changes in ANS patients before and 6 months posttreatment. The correlation between MoCA scores and cellular metabolism of hippocampus were analyzed.

**Results:**

There was no significant difference in MoCA scores among the three groups (*p* > 0.05). The NAA/Cr ratios in the bilateral hippocampus of ANS patients was significantly lower than those of the syphilis patients and control group (*p* < 0.05). The NAA/Cr ratios in the bilateral hippocampus was positively correlated with the MoCA scores (*r* = 0.4614, *p* = 0.0232; *r* = 0.4133, *p* = 0.0447); it was negatively correlated with the number of white blood cells and protein levels in cerebrospinal fluid (CSF) (*p* < 0.05). There was no significant difference in the cellular metabolism of hippocampus between the syphilis and control group (all *p* > 0.05). Compared with before treatment, ANS patients showed a significant improvement in NAA/Cr ratios at 6-month follow-up after high-dose penicillin treatment (*p* < 0.05), and no difference compared with the syphilis and control groups (*p* > 0.05).

**Conclusion:**

^1^H-MRS can effectively detect cellular metabolism changes in hippocampus of ANS patients. Although ANS patients have no obvious clinical symptoms, there are cellular metabolic changes in hippocampus and reduced neuronal metabolic function. Standardized treatment can correct this abnormal change.

## Background

Neurosyphilis is a infectious diseases caused by *Treponema pallidum* invading the central nervous system (CNS). Its clinical manifestations are complex and nonspecific (1). It can have clinical symptoms such as fever, headache, cognitive impairment, mental disorder, hemiplegia and epilepsy, or no obvious clinical symptoms (2, 3). Based on their clinical manifestations and invasion sites, neurosyphilis can be classified into asymptomatic type, interstitial type (meningeal and vascular), parenchymal type (optic atrophy and general paralysis of the insane) and tabes dorsalis (4–6). Neurosyphilis is difficult to diagnose in its early stages, mainly asymptomatic or with only mild headaches, and is prone to missed diagnosis; Neurosyphilis can occur at any stage of syphilis. Workowski et al. (7) found that about 20% of untreated syphilis patients will eventually develop into asymptomatic neurosyphilis, of which about 10% will develop into symptomatic neurosyphilis. Once it develops into severe types such as general paresis of insane (GPI) or tabes dorsalis, it will seriously affect the quality of life of patients and bring heavy burden to their families and society.

Previous studies have shown that the stability or improvement of clinical symptoms and the recovery of abnormal CSF indicators after early neurosyphilis treatment are significantly better than those in late neurosyphilis, especially asymptomatic neurosyphilis that can often achieve good therapeutic effects after standardized treatment (6, 8). Therefore, diagnosis of neurosyphilis timely and effective treatment of syphilis are crucial for improving patient prognosis. For the treatment of neurosyphilis, multiple guidelines recommend the use of high-dose penicillin as the preferred treatment (7, 9). Multiple studies also have shown that neurosyphilis is closely related to cognitive function, and commonly show up cognitive impairment (10–12). However, previous studies have mostly focused on the cognitive function of symptomatic neurosyphilis patients. As a common type of neurosyphilis, the change in cognitive function in ANS patients has received little attention. This may be related to the fact that ANS patients have no obvious clinical symptoms and are difficult for patients and clinicians to diagnose, resulting in less attention paid to cognitive function.

We know biochemical changes in many diseases occur earlier than clinical signs and macroscopic structural abnormalities. Magnetic resonance spectroscopy (MRS) can non-invasively detect metabolic and biochemical changes in living tissues and organs, and perform quantitative analysis of compounds, which helps to discover pathological changes that cannot be displayed by conventional morphological imaging. Currently, it has been widely used in the common diseases such as Alzheimer’s disease, mild cognitive impairment, Parkinson’s disease, etc. (13–16). Most symptomatic neurosyphilis patients have cognitive impairment, which attracts high attention from clinicians. However, due to the lack of obvious clinical symptoms, clinicians pay less attention to the cognitive function of ANS. But the application of MRS technology allows us to non-invasively study the changes in cellular metabolism of living brain tissue.

For that purpose, this study used the MoCA scale to evaluate the cognitive function of patients with ANS, and used ^1^H-MRS technology to detect the cellular metabolism of bilateral hippocampus in patients, exploring the cognitive function and changes in hippocampal cellular metabolism of ANS patients, as well as the changes in cognitive function and hippocampal cellular metabolism of ANS patients after high-dose penicillin treatment. The aim is to improve clinicians understanding of the cognitive function of ANS patients and provide reference for further early diagnosis and treatment.

## Materials and methods

### Cases and groups

Twenty-four patients with ANS and 20 patients with Syphilis who were admitted in Hangzhou Third people’s Hospital from June 2021 to July 2025 were selected as the research subjects. At the same time, 23 individuals who came for physical examinations at the same period were selected as the normal control (NC) group. All selected cases must meet the diagnostic criteria for syphilis and ANS (9, 17). The study was approved by the ethics committee of the Hangzhou Third people’s Hospital (No: 2023KA031).

The inclusion criteria for ANS are: (1) No obvious clinical neurological symptoms, (2) Positive CSF *T. pallidum* gelatin particle agglutination test (TPPA) and toluidine red unheated serum test (TRUST); (3) CSF proteins >50 mg/dL or CFS white blood cell counts ≥5 × 10^6^/L; (4) Exclude other causes of CNS infections.

The inclusion criteria for syphilis patients include a history of *T. pallidum* infection, positive serum TRUST and TPPA, presence/absence of evidence of syphilis damage in systematic examination, normal CSF examination (negative CSF TRUST and TPPA) through lumbar puncture, and exclusion of neurosyphilis diagnosis.

Exclusion criteria: (1) Age under 18 years; (2) Patients with incomplete clinical data in medical records; (3) Symptomatic neurosyphilis patients; (4) Individuals with positive blood HIV; (5) Patients with severe diseases of the heart, lungs, liver, kidneys, etc.; (6) Individuals with previous dementia or severe mental illness; (7) Penicillin allergy patients.

## Methods

### Collection of basic information of patients

It is a retrospective case study, we collect data on age, gender, years of education, and comorbidities of ANS and syphilis patients through the hospital’s electronic medical record system, collect general information of the control individuals simultaneously.

### CSF test

ANS and syphilis patients underwent lumbar puncture within 48 h of admission (ANS patients readmitted for lumbar puncture after 6 months of treatment). 5 mL of CSF was collected in a sterile tube and tested for white blood cell count, glucose, protein, chloride, ADA, LDH, as well as TPPA and TRUST titers.

### Assessment of cognitive function

The Montreal cognitive assessment (MoCA) was use to assess cognitive function. MoCA scale include 8 cognitive domains (visual spatial executive function, naming, memory, attention, language, abstraction, delayed recall and orientation), with a total score of 30 points. The higher the score, the better the cognitive function. A score of ≥26 is considered normal, while a score of <26 indicates cognitive impairment (for individuals with less than or equal to 12 years of education, 1 point will be added to the test results). All patients completed the cognitive function assessment within 48 h of admission, which was conducted by a trained neurologist.

### ^1^H-MRS technology for detecting cellular metabolism in bilateral hippocampus

**Figure 1:**
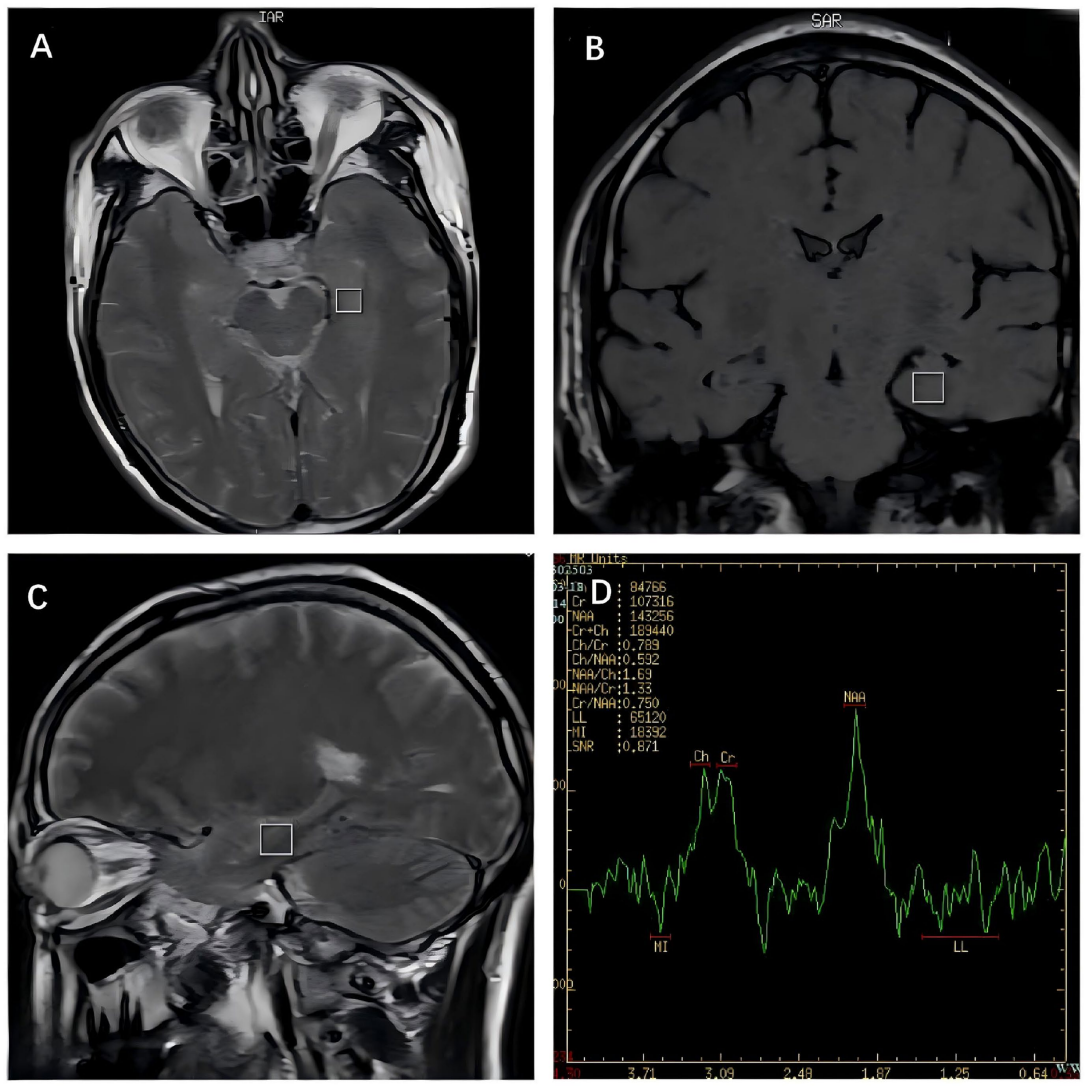
^1^H-magnetic resonance spectroscopy for the left hippocampal region: **(A)** axial position; **(B)** coronal localization; **(C)** sagittal position; **(D)** spectral diagram.

All subjects were examined using GE 1.5 T magnetic resonance scanner (GE Signa HDxt 1.5 T; GE Medical systems, LLC, USA) with a standard 8-channel head coil within 48 h of admission. All participants underwent the following examinations: (1) Routine scan: TSE sequence is used for routine MRI examination of the head (including T1 weighted image, T2 weighted image, fluid attenuated inversion recovery). (2) ^1^H-MRS detection sequence: Perform T1WI, T2WI localization scans in axial, sagittal and coronal positions, and ^1^H-MRS using multi voxel spectroscopic techniques such as Chemical Shift Imaging (CSI) sequences and Point Resolved Spectroscopy (PRESS) analysis, with a repetition time of 2000 ms, echo time of 35 ms, 128 acquisition times. Obtain signal spectra through gradient selection combined with phase encoding. At the same time, the Chemical Shift Selective Saturation (CHESS) method is used for water suppression, and the operator manually homogenizes to optimize magnetic field uniformity, ensuring that the full width at half maximum (FWHM) of the homogenized field is less than 10 Hz. (3) The region of interest is located in the left and right hippocampus, accurately positioned in three directions: axial, sagittal and coronal localization (Figure 1), with a voxel size of 20 mm × 20 mm × 10 mm. The post-processing of the spectrogram is completed using the Function tool (SAGE/GE Healthcare) software package that comes with the scanner. The processing flow includes: The 4 Hz exponential window function is used for filtering, Fourier transform, automatic phase correction and baseline correction. Fit the area under the metabolite peak based on the Gaussian-Lorentzian mixture model. The scanner automatically completes baseline calibration and metabolite identification, and calculates the ratios of N-acetylaspartate (NAA)/creatine (Cr), choline (Cho)/Cr and NAA/Cho in the bilateral hippocampus of the subjects. All MRS examinations were performed by experienced radiologists who were blind to the subject’s diagnosis.

### Spectral quality control standards

The spectra included in the analysis must met the following criteria: (1) FWHM < 10 Hz; (2) Signal to Noise Ratio (SNR) > 5; (3) No obvious motion artifacts or lipid contamination; (4) The Cramér-Rao lower bound (CRLB) evaluates the uncertainty of metabolite fitting, and the CRLBs of metabolites (NAA, Cho, Cr) are all below 20%. Any spectra that do not met these criteria are excluded.

### Treatment and follow-up

ANS patients were given intravenous infusion of penicillin sodium (24 million units/d); Once/4 h, 4 million units each time, for 14 consecutive days. After treatment, 2.4 million units of benzylpenicillin G will be administered via intramuscular injection once a week for 3 weeks. For patients receiving their first penicillin treatment, take 20 mg of prednisone orally once a day for 3 days before treatment to prevent Jarisch–Herxheimer reaction (18). Patients with stage I, II syphilis, and early latent syphilis were given 2.4 million units of benzylpenicillin G via intramuscular injection once a week, for a total of one dose; Late latent syphilis patients were given 2.4 million units of benzylpenicillin G, once a week, for a total of 3 times.

Patients with ANS should undergo lumbar puncture re-examination 6 months after their treatment. If the white blood cell count in the CSF does not decrease or the TRUST titers in the CSF does not decrease to 1/4 of its original level after treatment (or if the initial titers is less than 1:2, the TRUST in the CSF has not turned negative at this time), it is recommended to continue treatment; If the follow-up CSF test shows an increase in white blood cell count or a 4-fold increase in TRUST titer, further treatment is also necessary.

### Statistical analysis

SPSS 25.0 software was used for data processing and statistical analysis. Kolmogorov–Smirnov test was used to test the normality of each group of data. Measurement data that followed a normal distribution were represented as mean ± standard deviation, while abnormal distribution data were described as median (interquartile range) [M (Q1–Q3)]. The comparison between the two groups was performed using rank sum test; One way analysis of variance (ANOVA) was used to compare the means between multiple groups, while S-N-K method was used to test the comparison between two groups. Count data are expressed in frequency, and comparison between groups is performed using chi square test or Fisher’s exact test. We used Pearson correlation analysis for normally distributed data, while Spearman correlation analysis for abnormally distributed data. A two-sided test with *p* < 0.05 was used to determine statistical significance.

## Results

### Baseline characteristics of patients in different groups

According to the inclusion and exclusion criteria, this study successfully included 24 cases of ANS patients, including 19males and 5 females, with an average age of 50.5 ± 13.0, years of education 10.3 ± 3.6 years. The comorbidities included hypertension in 5 cases, diabetes in 2 cases, and tumors in 2 cases; there were 20 patients in the syphilis group, including 13 males and 7 females, with an average age of 42.7 ± 17.3 and an average education duration of 11.4 ± 3.2 years. Comorbidities: 4 cases of hypertension, 1 case of diabetes, and 1 case of tumor. 23 cases in the NC group, 13 males and 10 females, with an average age of 52.0 ± 15.9 and years of education of 10.8 ± 2.1. There were 8 cases of hypertension, 3 cases of diabetes, and 2 cases of tumor. There were no significant statistical differences in age, sex, and years of education among the three groups (*p* > 0.05). Table 1. Compared with syphilis group, the CSF white blood cell counts, protein, and ADA content in the ANS group were significantly increased (*p* < 0.01; *p* < 0.05), Table 1.

**Table 1:** Comparison of baseline characteristics among three groups.

Characteristics	NC group (*n* = 23)	Syphilis group (*n* = 20)	ANS group (*n* = 24)	F/x^2^/t/z	*p*
Age (years)	52.0 ± 15.9	42.7 ± 17.3	50.5 ± 13.0	2.226	0.1163
Gender (male/female) (cases)	13-Oct	13-Jul	19-May	2.7914	0.2477
Years of education (years)	10.8 ± 2.1	11.4 ± 3.2	10.3 ± 3.6	0.7156	0.4928
Comorbidity (cases)					
Hypertension	8	4	5	1.6416	0.4401
Diabetes	3	1	2	0.8666	0.6484
Tumor	2	1	2	0.2526	0.8813
CSF					
White blood cell counts (×10^6^/L)	NA	2.0 (1.0,3.0)	27.5 (8.5,65.3)	3.254	0.0023
Protein (mg/dl)	NA	40.2 ± 11.0	52.7 ± 18.7	2.517	0.0159
Chlorine (mmol/L)	NA	126.4 ± 3.3	127.0 ± 2.9	0.6202	0.5387
Glucose (mmol/L)	NA	3.3 ± 0.4	3.5 ± 0.8	1.145	0.2593
LDH (U/L)	NA	24.4 ± 5.5	25.1 ± 7.1	0.3663	0.7162
ADA (U/L)	NA	0.7 ± 0.5	1.1 ± 0.5	2.1856	0.0239

NC, normal control; ANS, asymptomatic neurosyphilis; NA, not applicable; CSF, cerebrospinal fluid; LDH, lactate dehydrogenase; ADA, adenosine deaminase.

### Cognitive function analysis

The MoCA scores of the three groups of patients were 27.6 ± 1.8 vs. 27.8 ± 2.1 vs. 28.0 ± 1.8; There was no difference (*p* > 0.05); Among the three groups, there were 2, 3, and 3 patients with MoCA scores below 26 points respectively, and there was no statistically significant difference in the incidence of cognitive impairment (*p* > 0.05) Table 2.

**Table 2:** Comparison of MoCA scores between the 3 groups.

Cognitive measures	Control group (*n* = 23)	Syphilis group (*n* = 20)	ANS group (*n* = 24)	F	*p*
Visual spatial executive function	4.7 ± 0.6	4.7 ± 0.7	4.3 ± 0.9	1.3420	0.2686
Naming	2.9 ± 0.3	2.9 ± 0.3	3.0 ± 0.2	0.5703	0.5682
Attention	4.8 ± 1.0	5.1 ± 1.1	5.4 ± 0.8	2.3020	0.1083
Language	3.0 ± 0.2	3.0 ± 0.2	2.9 ± 0.3	0.6733	0.5136
Abstraction	2.0 ± 0.2	2.0 ± 0.2	1.9 ± 0.3	0.3925	0.6770
Delayed recall	4.7 ± 0.5	4.4 ± 0.6	4.6 ± 0.6	1.7570	0.1807
Orientation	5.7 ± 0.5	5.9 ± 0.3	5.9 ± 0.3	1.9150	0.1557
MoCA total scores	27.6 ± 1.8	27.8 ± 2.1	28.0 ± 1.8	0.3746	0.6890

ANS, asymptomatic neurosyphilis; MoCA, Montreal cognitive assessment.

### Changes in cellular metabolism in bilateral hippocampus in three groups

**Figure 2:**
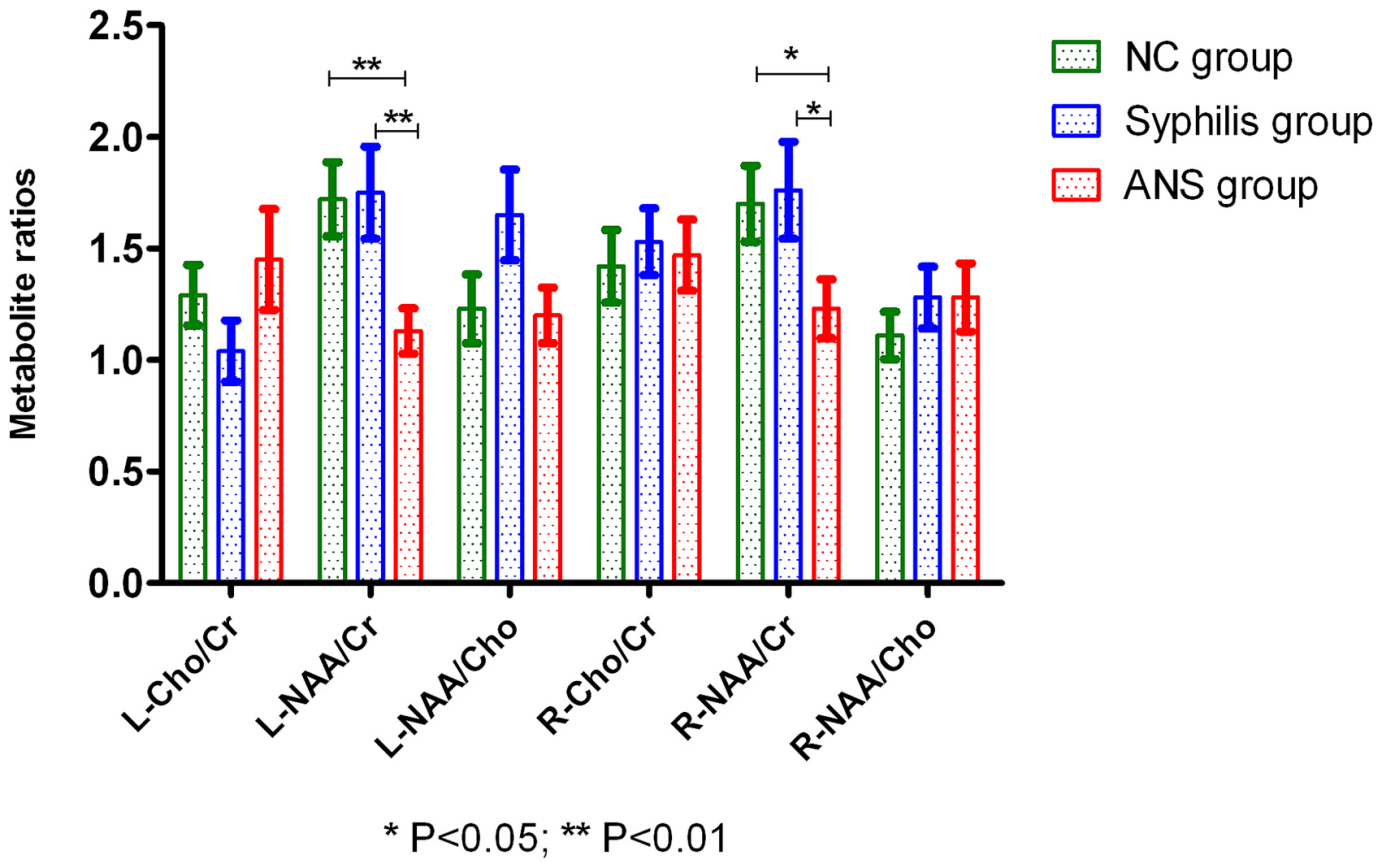
The NAA/Cr ratios in the bilateral hippocampus of the ANS group were significantly lower than those in the syphilis and NC group (*p* < 0.05; *p* < 0.01).

There was a significant difference in the ratios of NAA/Cr in the bilateral hippocampus of the three groups (*p* < 0.01, *p* < 0.05). The NAA/Cr ratios in the bilateral hippocampus of the ANS group were significantly lower than those in the syphilis group and the NC group (*p* < 0.01; *p* < 0.05), while there was no significant statistical difference in the NAA/Cr ratios between the syphilis and NC group (*p* > 0.05). There was no significant difference (*p* > 0.05) in Cho/Cr and NAA/Cho ratios in the bilateral hippocampal regions among the three groups. Table 3 and Figure 2.

**Table 3:** Comparison of hippocampal cellular metabolism among three groups with ^1^H-MRS.

Metabolic measures	Control group (*n* = 23)	Syphilis group (*n* = 20)	ANS group (*n* = 24)	F	*p*
L-Cho/Cr	1.29 ± 0.65	1.04 ± 0.61	1.45 ± 1.11	0.3767	0.6878
L-NAA/Cr	1.72 ± 0.80	1.75 ± 0.92	1.13 ± 0.50	5.2260	0.0080
L-NAA/Cho	1.23 ± 0.74	1.65 ± 0.91	1.20 ± 0.61	1.3990	0.2551
R-Cho/Cr	1.42 ± 0.78	1.53 ± 0.67	1.47 ± 0.78	0.1170	0.8898
R-NAA/Cr	1.70 ± 0.82	1.76 ± 0.97	1.23 ± 0.65	2.9350	0.0407
R-NAA/Cho	1.11 ± 0.51	1.28 ± 0.62	1.28 ± 0.75	0.5750	0.5659

ANS, asymptomatic neurosyphilis.

### Changes in cognitive function, cellular metabolism in hippocampus, and CSF/blood indicators in patients with ANS before and after 6 months post treatment

24 ANS patients underwent 6 months follow-up after high-dose penicillin treatment. Compared to before treatment, the NAA/Cr ratios in the bilateral hippocampus of ANS patients were significantly increased after 6 months posttreatment (*p* < 0.01). The white blood cell count, protein, and ADA content in CSF were significantly reduced (*p* < 0.01), while the NAA/Cr ratios in the bilateral hippocampus were not significantly different from those in the syphilis and NC group (*p* > 0.05). The number of cases with blood TRUST titers greater than 1:8 significantly decreased (*p* < 0.01); however, there were no significant difference in Cho/Cr, NAA/Cho ratios, as well as CSF glucose, chloride, and LDH concentrations (*p* > 0.05) Table 4.

**Table 4:** Comparison of cellular metabolism in hippocampus, CSF, and blood TRUST titers in 24 ANS patients between before treatment and 6 months follow-up.

Biomarker indexes	Before treatment	6 months post treatment	t/z/x^2^	*p*
L-Cho/Cr	1.45 ± 1.11	1.56 ± 0.48	0.4284	0.6732
L-NAA/Cr	1.13 ± 0.50	1.79 ± 0.71	3.795	0.0009
L-NAA/Cho	1.20 ± 0.61	1.01 ± 0.56	1.021	0.3202
R-Cho/Cr	1.47 ± 0.78	1.60 ± 0.82	0.9437	0.3572
R-NAA/Cr	1.23 ± 0.65	1.76 ± 0.78	3.954	0.0006
R-NAA/Cho	1.28 ± 0.75	1.36 ± 0.60	0.4889	0.6305
CSF				
White blood cell counts (×10^6^/L)	27.5 (8.5,65.3)	2.5 (1.0,7.8)	3.608	0.0015
Protein (mg/dl)	52.7 ± 18.7	41.4 ± 6.6	2.872	0.0086
Chlorine (mmol/L)	127.0 ± 2.9	126.2 ± 3.4	0.7528	0.4595
Glucose (mmol/L)	3.5 ± 0.8	3.4 ± 0.5	1.172	0.2538
LDH (U/L)	25.1 ± 7.1	23.3 ± 5.1	1.722	0.0998
ADA (U/L)	1.1 ± 0.5	0.7 ± 0.4	3.552	0.0019
Blood TRUST titer (case)				
≤1:8	18	9	6.8571	0.0088
>1:8	6	15		

CSF, cerebrospinal fluid; LDH, lactate dehydrogenase; ADA, adenosine deaminase; TRUST, toluidine red unheated serum test.

### Correlation analysis

**Figure 3:**
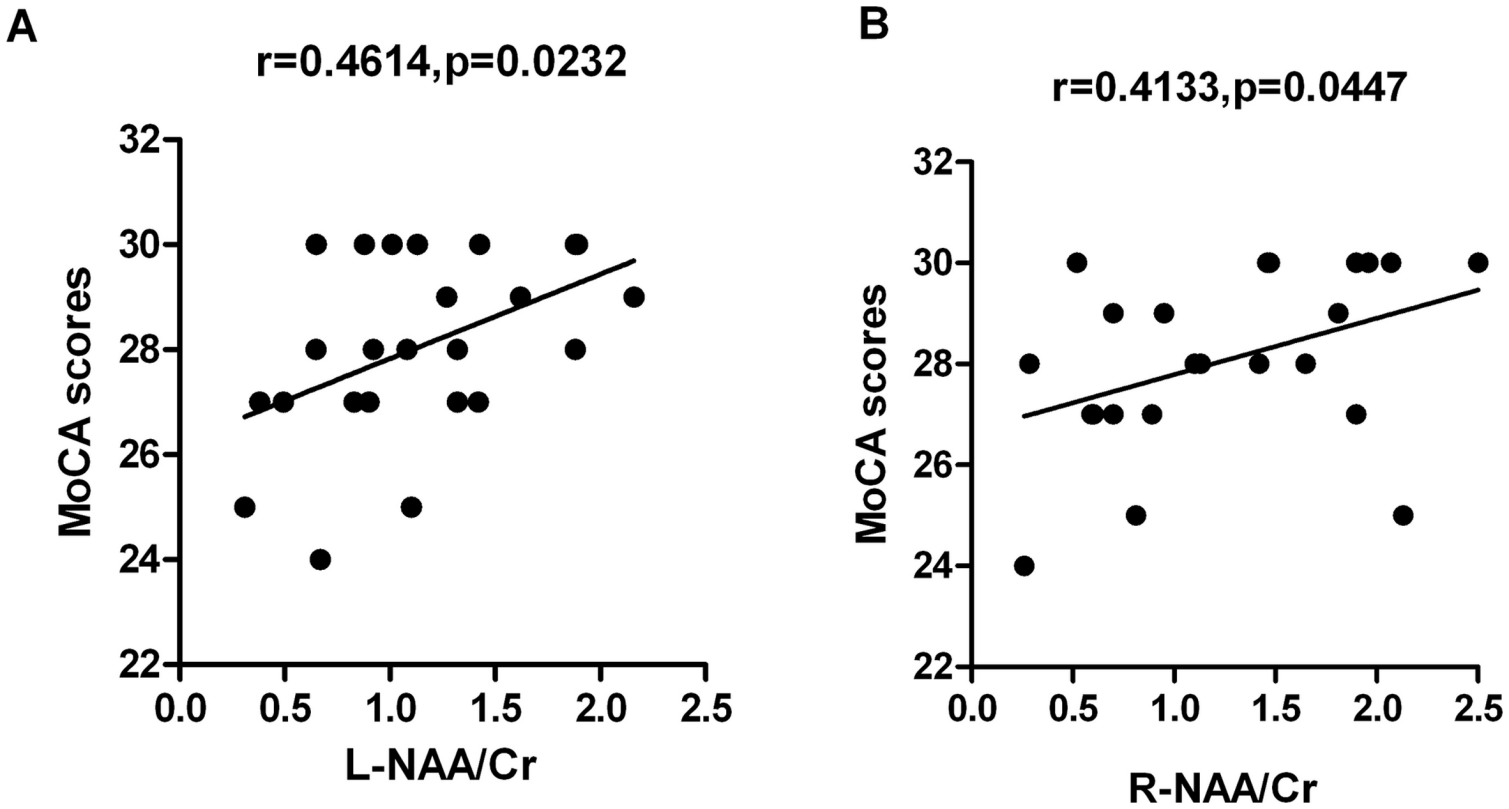
**(A)** The MoCA scores of ANS patients was positively correlated with the NAA/Cr ratios of left hippocampus, *r* = 0.4614, *p* = 0.0232; **(B)** The MoCA scores was positively correlated with the NAA/Cr ratios of right hippocampus, *r* = 0.4133, *p* = 0.0447.

**Figure 4:**
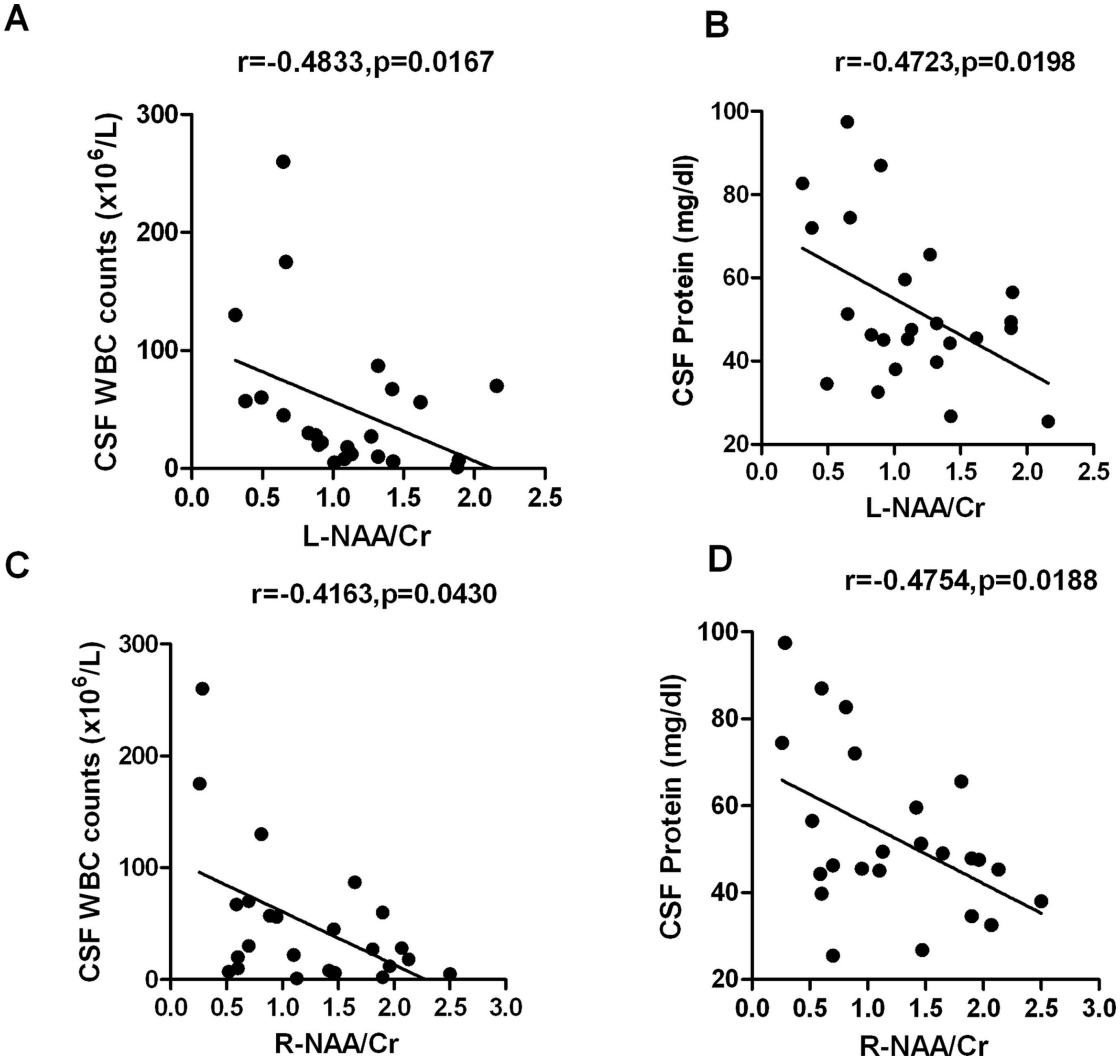
The ratios of NAA/Cr in the left/right hippocampus of ANS patients were negatively correlated with the number of white blood cells and protein levels in the CSF. **(A)** NAA/Cr in the left hippocampus vs. CSF white blood cells, *r* = −0.4833, *p* = 0.0167; **(B)** NAA/Cr in the left hippocampus vs. CSF proteins, *r* = −0.4723, *p* = 0.0198; **(C)** NAA/Cr in the right hippocampus vs. CSF white blood cells, *r* = −0.4163, *p* = 0.0430; **(D)** NAA/Cr in the right hippocampus vs. CSF proteins, *r* = −0.4754, *p* = 0.0188.

The MoCA scores of ANS patients was positively correlated with the NAA/Cr ratios of bilateral hippocampus (*r* = 0.4614, *p* = 0.0232; *r* = 0.4133, *p* = 0.0447), but there was no significant correlation between MoCA scores and Cho/Cr and NAA/Cho ratios (*p* > 0.05). Further analysis revealed that the ratios of NAA/Cr in the left/right hippocampus of ANS patients were negatively correlated with the number of white blood cells and protein levels in the CSF (*p* < 0.01) Figures 3, 4.

## Discussion

Syphilis is a sexually transmitted disease caused by infection with *T. pallidum*. While, neurosyphilis is a CNS infectious disease resulting from *T. pallidum* invasion of the CNS, causing damage to the meninges, brain and spinal cord. It can occur at any stage of *T. pallidum* infection and is classified as symptomatic neurosyphilis or ANS based on the presence or absence of symptoms. In recent years, with the rising incidence of syphilis infection, the prevalence of neurosyphilis has also shown an increasing trend (19–21). Studies report that approximately 10%–20% of untreated syphilis patients ultimately develop to neurosyphilis (7). Neurosyphilis has a variety of clinical manifestations and few specific symptoms, making it highly prone to missed diagnosis. Once the CNS is affected, it may result in early functional impairment or late irreversible organic abnormalities, even life-threatening. The prognosis of late treatment is poor, whereas in early stage, especially ANS patients, has the possibility of cure after standardized treatment. Therefore, clinicians should prioritize the early diagnosis and treatment of neurosyphilis to improve their outcomes. The diagnosis of neurosyphilis requires comprehensive consideration of clinical manifestations, syphilis serology and CSF changes, cannot be determined based on a single laboratory test indicator. All ANS patients included in this study demonstrated positive TRUST and TPPA test in serum/cerebrospinal fluid, elevated white blood cell counts and protein levels in CSF, with no obvious clinical neurological symptoms, meeting the diagnostic criteria for asymptomatic neurosyphilis.

The ANS patients included in this study showed no significant cognitive impairment, with no marked differences in MoCA scores compared to the syphilis and NC groups. This aligns with the characteristic of ANS patients exhibiting no obvious clinical neurological symptoms, posing challenges for clinicians in early identification of ANS. After *T. pallidum* invades the CNS, it can cause chronic inflammation. This leads to various symptoms due to the destruction of neurons in the affected areas, cognitive impairment being one of the common clinical manifestations in neurosyphilis patients. When *T. pallidum* attacks the CNS, it invades small blood vessels in the temporal lobe, damages vascular walls, alters vascular permeability, and induces localized ischemic changes in brain tissue. This leads to pathological manifestations such as neuronal loss accompanied by glial proliferation, which may be associated with the onset and progression of cognitive impairment (22).

Magnetic resonance spectroscopy (MRS) is a non-invasive imaging technique that reveals the metabolic status of living tissue. By detecting changes in the concentration and ratio of various metabolites within brain tissue of interest, it measures cellular metabolic alterations, enabling evaluation of tissue changes from a brain metabolic perspective even before morphological alterations occur. MRS technology has been extensively applied in the study of neurological and psychiatric disorders, with the most commonly measured metabolites being NAA, Cr, Cho, and their relative ratios (13–16). MRS technology has been extensively applied in neurosyphilis patients (12, 23). For instance, Che et al. (12) employed ^1^H-MRS to evaluate hippocampal cellular metabolic changes in 80 patients with GPI and 57 healthy controls, they found significantly reduced the ratios of NAA/Cr and NAA/Cho in the bilateral hippocampus of GPI, indicating reduced neuronal integrity/metabolic function. Using the same technique, this study assessed hippocampal cellular metabolism in ANS patients. Compared with controls and syphilitic patients, ANS patients showed significantly reduced bilateral hippocampus NAA/Cr ratios despite comparable MoCA scores across all three groups. NAA/Cr ratios positively correlated with MoCA scores, while further analysis revealed negative correlations between NAA/Cr ratios and CSF white blood cell counts and protein levels. ANS patient’s exhibit reduced neuronal integrity/metabolic function in the hippocampal region despite lacking early clinical manifestations of dementia and maintaining normal MoCA scores. This may correlate with the pathological changes of neurosyphilis, primarily characterized by inflammatory cell reactions, lymphocyte and plasma cell infiltration of cerebral cortical microvessels. This study also found no statistically significant differences in bilateral hippocampus NAA/Cr, Cho/Cr, or NAA/Cho levels between the syphilis and the control group. It suggests that patients with syphilis, *T. pallidum* has not yet invaded the CNS, if actively treated in the early stage, there may not lead to damage to CNS.

Since penicillin was first used to treat syphilis, penicillin preparations have remained the first-line drug recommended by multiple guidelines for treating syphilis/neurosyphilis. In this group of ANS patients, 6 months follow-up after high-dose penicillin therapy showed significant decreases in CSF white blood cell counts, protein levels, and blood TRUST titers, tending toward normal, indicating treatment efficacy. Moreover, bilateral hippocampus NAA/Cr ratios gradually increased, showing no statistically significant difference compared to the control group. This may be related to the correction of abnormal cellular metabolism after therapy. Previous studies have also demonstrated that penicillin treatment can improve cognitive function to a certain extent in neurosyphilis patients (24–27). Davis et al. (10) analyzed 96 individuals with syphilis at high risk of CSF-defined neurosyphilis, they found that two-thirds of patients exhibited cognitive impairment, which was 3.8 times higher in those with CSF white blood cell counts >5/μL compared to those with ≤5/μL, and cognitive impairment improved after treatment. Decreased NAA/Cr ratios in bilateral hippocampus of ANS patients may be related to local inflammatory response and mitochondrial dysfunction caused by *T. pallidum* infection, which leading to reduced NAA synthesis and the damage to the neuronal cytoskeleton and axonal transport. In addition, the improvement of NAA/Cr ratios in ANS patients after high-dose penicillin treatment, it further supports that this abnormality is largely functional and reversible, rather than permanent structural damage. In this study, MoCA scores remained unchanged after high-dose penicillin therapy in included ANS patients. This discrepancy may stem from our cohort comprising ANS patients, differing from previous studies’ inclusion criteria. However, MRS revealed correction of abnormal hippocampal cellular metabolism in this group after high-dose penicillin therapy. Based on this, we speculate that ^1^H-MRS assessment of hippocampal cellular metabolism holds potential reference value for diagnosing and evaluating treatment efficacy of neurosyphilis.

This study has certain limitations. First, it is a single-center, retrospective study with a small sample size of ANS patients. This is primarily due to our strict inclusion criteria (excluding some ANS patients with incomplete data and symptomatic neurosyphilis). We conducted intergroup comparisons and correlation tests on multiple hippocampal metabolite ratios, MoCA scores and CSF markers, but did not use methods such as false discovery rate (FDR) to correct for the risk of false positives caused by multiple comparisons. Large-scale, multi-center, prospective studies are needed to further validate these findings in future. Second, this study uses Cr as a reference, although previous many studies have suggested that Cr content is relatively stable in all brain metabolites and is generally used as a reference for standardizing metabolites in the body, due to various factors may affect Cr metabolism, the difference may be smaller if water reference or absolute quantification is used. At the same time, there was lack of segmentation and correction of tissue components within voxels (gray matter/white matter/cerebrospinal fluid), this may lead to small differences between groups, potentially affecting metabolite ratios, and future research should avoid this limitation. Additionally, our stduy was unable to fully control for the impact of potential confounding factors on hippocampal metabolism. Although there is no statistical difference among the three groups in age, gender, years of education and comorbidities (hypertension, diabetes etc.), due to the limited sample size, we cannot rule out these comorbidities, especially their unquantified severity, which may have a mixed effect on hippocampal metabolic ratio. The hippocampal metabolic abnormalities discovered in this study may also be the result of the combined effects of asymptomatic neurosyphilis and these vascular risk factors. Future research requires multivariate regression analysis with larger samples or strict matching of comorbidities at enrollment to more accurately distinguish the independent effects of *T. pallidum* infection. Finally, the follow-up period for ANS patients was relatively short, lasting only 6 months. Previous studies have shown that high-dose penicillin treatment can improve cognitive function of neurosyphilis in the short term, but long-term outcomes remain poor (27). Extending the follow-up period for patients and integrating changes in their clinical data before and after standardized treatment may yield more meaningful findings.

In summary, ^1^H-MRS effectively detects cellular metabolic alterations in hippocampus of ANS patients. Despite lacking overt clinical symptoms, ANS patients exhibit cellular metabolic changes and reduced neuronal integrity/metabolic function, these abnormalities can be corrected following standardized treatment. While, syphilis patients do not yet exhibit metabolic abnormalities in hippocampus. For syphilis patients, if ^1^H-MRS examination reveals a decrease of NAA/Cr ratios in the hippocampus, clinicians should be aware that the patient may have progressed to the stage of neurosyphilis and should undergo further examination and treatment timely. Therefore, ^1^H-MRS technology to detect the cellular metabolism of hippocampus in ANS patients has certain clinical value for the early diagnosis and prognosis assessment.

## Data Availability

The raw data supporting the conclusions of this article will be made available by the authors, without undue reservation.
